# Colorimetric Diagnostic Capillary Enabled by Size Sieving in a Porous Hydrogel

**DOI:** 10.3390/bios10100130

**Published:** 2020-09-23

**Authors:** John Mello Camille C. Guzman, Sheng-Min Hsu, Han-Sheng Chuang

**Affiliations:** 1 Department of Biomedical Engineering, National Cheng Kung University, Tainan 701, Taiwan; p86077038@ncku.edu.tw; 2 Department of Ophthalmology, National Cheng Kung University Hospital, Tainan 701, Taiwan; shengmin@mail.ncku.edu.tw; 3 Center for Micro/Nano Science and Technology, National Cheng Kung University, Tainan 701, Taiwan

**Keywords:** porous hydrogel, immunoassay, biomarker, lipocalin 1, colorimetry, gold nanoparticles

## Abstract

Handy and disposable point-of-care diagnostics facilitate the early screening of severe diseases in resource-limited areas. To address urgent needs in inconvenient sites, a simple colorimetric diagnostic device equipped with a capillary tube with porous hydrogel and immunocomplex particles was developed for the rapid detection of biomarkers (16 min). In this device, probe particles attach to capture particles (dp = 40 µm) and form sandwiched immunocomplexes in the presence of target biomarkers, and a red color progressively emerges when the sandwiched immunocomplex particles are blocked by the porous hydrogel embedded inside the glass capillary. Colorimetric aggregation was recorded using a smartphone and analyzed with imaging software. The limit of detection reached 1 ng/mL and showed a maximum of 79% accuracy compared with that obtained through a conventional spectrophotometric technique. The level of a diabetic retinopathy (DR) biomarker, lipocalin-1 (LCN-1), was measured in 1 µL of a human tear sample and used in testing the practicability of the proposed device. All healthy subjects showed lower intensity levels than the other diabetic counterparts (proliferative DR or nonproliferative DR patients), implying the potential of this device in clinical applications. Overall, the diagnostic device facilitates point-of-care-testing and provides a low-cost (~1 USD), compact, and reliable tool for early diagnosis in resource-limited areas.

## 1. Introduction

Epidemics have been threatening public health for centuries. The battle has become increasingly fierce recently as the highly contagious disease, COVID-19, broke out in early 2020 and rapidly spread worldwide, raising an unprecedented crisis [1,2]. Although early diagnosis is an effective measure for containing mass transmission, heavy dependence on high-end instruments forms a barrier to facile realization [3]. The consequences impact healthcare systems, especially in resource-limited areas [4].

To deal with these challenges, microfluidic technology has evolved since its advent in the 1990s. It is a research frontier and a possible alternative for sophisticated instruments. This technology has demonstrated the feasibility of device miniaturization and the development of new art in the Lab-on-Chip (LoC) system for various chemical and biological applications [5]. LoC has emerged as a powerful tool in the field of biomedical engineering. In contrast to their macroscale counterparts, LoC devices have many advantages, including small volumes of sample/reagent (down to the nanoliter), fast throughput, low cost, operational simplicity, and good potential for disposability [6,7,8]. In the manufacture of an LoC device, one of the most important considerations is material selection. Different materials have been used in microfluidic platforms, such as silicon [9], glass [10,11], polymer like polydimethylsiloxane and poly(methyl methacrylate) [12,13,14,15] and paper [16,17,18,19]. Most of these materials are not well suited to point-of-care testing (POCT) applications for low-resource settings, such as developing nations. Nevertheless, paper has shown desirable results in analytical and clinical chemistry for a decade. Paper has been used as a substrate in analytical testing, but paper-based assays usually show only a positive or negative result in most cases. Thus, despite offering quantitative data, they cannot detect low-level concentration when samples with microlevel volumes are used, such as testing strips for pH, pregnancy, and diabetes [20]. Therefore, paper-based assays are still limited in terms of producing reliable tools for POCT diagnostics.

Currently, researchers are exploring novel approaches for addressing some of the limitations of paper-based assays in disease diagnosis at a POCT setting [21]. POCT can be implemented by untrained individuals at any location, allowing timely treatment [22]. The collection and analysis of samples, such as whole blood, urine, saliva, pathogens, and proteins, for diagnosis and disease management in hospitals, clinics, or even homes is therefore possible [23,24,25,26,27], based on the concept of POCT, Chin et al. [28] developed a new strategy that integrates an easy-to-use assay into a microfluidic device to replicate all ELISA steps in remote settings. Recently, Wang et al. [29] carried out POCT by developing microbeads conjugated with fluorescence-tagged probe antibodies on their microfluidic system for the early detection of diabetic retinopathy (DR). Existing techniques have issues about execution and assembly in large stationary laboratories [30]. For simplicity, researchers have recently shifted their focus to developing various smart phone-based detection systems for handy POCT applications. Smartphone cameras can be used in identifying color variations in colorimetric assays. With this capability, smartphone photography and image analysis are adopted to read the value of solution color for the quantitative analysis of urinary glucose [31]. In another study, Oncescu et al. [32] successfully demonstrated the applicability of smartphones to sweat and saliva biomarker detection.

**Figure 1:**
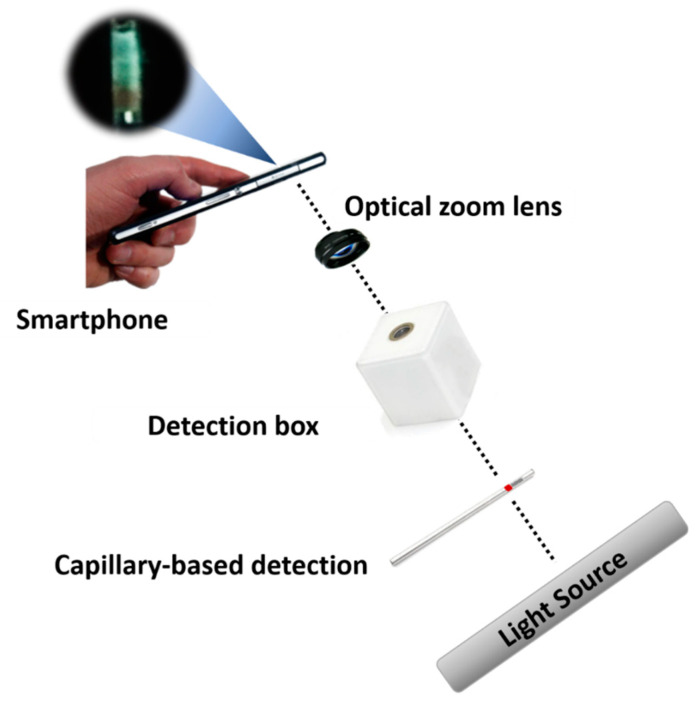
Illustration of an integrated platform consisting of a modified capillary device, a sandwiched immunocomplex and a portable detection system.

Given the popularity of POCT and smartphones in modern times, we proposed an integrated diagnostic platform equipped with a modified capillary-based device, a sandwiched immunocomplex, and a portable detection system for the rapid detection (16 min) of biomarkers in early disease diagnosis or low-abundance analytes (1 pg/mL) (Figure 1). A porous hydrogel was fabricated inside the microchannel through sol−gel chemistry. For the preparation of immunoassay, capture particles (d_p_ = 40 µm) and gold nanoparticles (AuNPs) as probe particles were bound using sandwiched immunocomplexes. For a proof of concept, LCN-1 was selected as a biomarker in the preparation of the sandwiched immunocomplexes. Liquid samples containing LCN-1 were firstly mixed with the capture and probe particles, and then injected into a modified capillary that filled with a porous hydrogel for color development. Subsequently, a smartphone was used to record the images for quantitative analysis. The limit of detection (LOD) reached 1 ng/mL. Only a small drop of a sample, as low as 1 μL, was required for effective measurement. The practicability of the proposed device was demonstrated by measuring LCN-1 in tear samples collected from seven volunteers. All healthy subjects showed considerably lower intensities, implying that their LCN-1 concentrations were markedly lower than those of the other clinically confirmed proliferative DR (PDR) and nonproliferative DR (PDR) patients. To a large extent, the proposed diagnostic platform shows great potential in the advancement of reliable diagnostic tools. The integration of this platform into smartphones and their applications for lens-less imaging will enable rapid disease diagnosis in resource-constrained areas.

## 2. Materials and Methods

### 2.1. Reagents

All solvents and chemicals were of reagent quality. Poly(ethylene glycol) diacrylate (PEGDA) Mn 250, 2-hydroxy-2-methylpropiophenone 97%, 2-(N-morpholino) ethanesulfonic acid (M3671), and N-hydroxysuccinimide (6066-82-6) were purchased from Sigma–Aldrich, St. Louis, MO, USA. Monoclonal Anti-LCN1 IgG (a7611) and a gold conjugation kit (ab154873) were purchased from Abcam, Cambridge, UK. Polyclonal anti-LCN1 IgG (17900-1-AP) was purchased from Proteintech. 1-(3-dimethylaminopropyl)-3-ethylcarbodiimide hydrochloride 98+% (A10807) was purchased from Alfa Aesar, Ward Hill, MA, USA. AuNPs (40 nm, 20 OD, C12-25-980-TA-50) were purchased from Nanopartz Inc., Loveland, CO, USA. Amine-modified polystyrene (PS) particles (40 µm, 01-01-404) were purchased from Micromod Partikeltechnologie GmbH, Germany and used in capturing AuNPs. Plain PS particles (45 µm) were purchased from Polysciences Asia Pacific, Inc., Taiwan for the fabrication of porous hydrogels.

### 2.2. Assembly of the Diagnostic Capillary with a Porous Hydrogel

**Figure 2:**
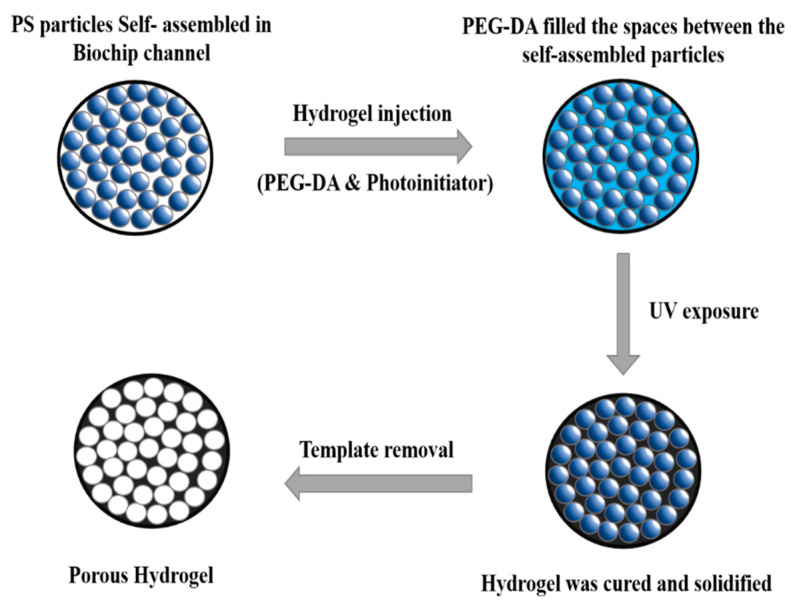
Schematic representation of the step-by-step formation of porous hydrogel.

For the fabrication of a porous hydrogel (Figure 2), a plain PS particle suspension was injected into a capillary tube and dried in a ventilated hood at 30 °C for 4 h. Surface tension gradually drove the particles to form a hexagonal close-packed structure during evaporation. Subsequently, 2 µL of 99 wt% PEGDA Mn 250 and 1 wt% 2-hydroxy-2-methylpropiophenone mixture was injected into the same capillary tube to fill the interstitial spaces within the self-assembled PS particle scaffold. After positioning the capillary vertically for 5 min, the hydrogel was photo-polymerized with a 312 nm UV lamp (EBF-280C, ALT) for 10 min. Excess capillary was cut for subsequent particle dissolution by the solvent. The capillary with the self-assembled structure was soaked in toluene and agitated in a shaker for 4 h for the removal of the PS particles. The treated hydrogel was later soaked in 0.1 M acetone for 1 h and then rinsed with deionized water three times for the removal of residual PS particles in the hydrogel. The porous hydrogel was relocated into another sterilized capillary tube (Supplementary Material Section S1 and Figures S1 and S2). In addition, a sponge plug was inserted into the new capillary tube and positioned next to the porous hydrogel to induce self-driving background flow. Given that colorimetric intensity may be influenced by sample volume, a mark was set on the capillary tube to define the appropriate sample volume to intake.

### 2.3. Preparation of Sandwiched Immunocomplex

#### 2.3.1. Functionalization of Capture Particles

**Figure 3:**
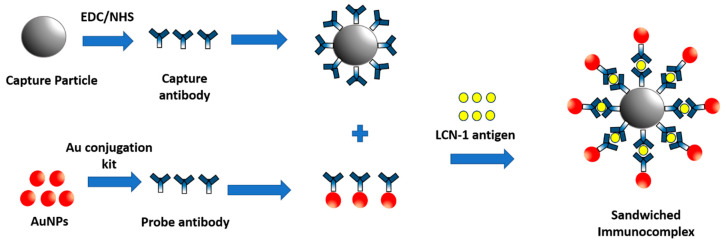
Illustrations for preparing sandwiched immunocomplex and detection process.

In this study, 40 µm amine-modified PS particles were used in preparing the capture particles. Successful sieving by the porous hydrogel was ensured by setting capture particle diameter close to the pore size. For particle surface treatment, 2-(N-morpholino) ethanesulfonic acid (M3671, Sigma, St. Louis, MO, USA) was used in centrifugally washing 20 µL of the selected particle twice. Unexpected aggregation was prevented by vortexing and sonicating the particle suspension in a sonicator for 30 min. Meanwhile, 2 µL of 10 mg/mL 1-ethyl-3-(3-dimethylaminopropyl)-carbodiimide (A10807, Alfa Aesar, Ward Hill, MA, USA), 4 µL of 10 mg/mL N-hydroxysuccinimide (6066-82-6, Sigma, St. Louis, MO, USA), and 10 µL of 50 mM (pH = 5.5) 2-(N-morpholino) ethanesulfonic acid (M3671, Sigma, St. Louis, MO, USA) buffer were added to 4.5 µL of 0.5 µg/µL mouse monoclonal anti-LCN-1 immunoglobulin G (IgG) (a76611, Abcam, Cambridge, UK) to activate the carboxyl groups on the capture antibody. Lastly, the antibody solution and the particle suspension were mixed and incubated in a thermal shaker at 800 rpm and 4 °C for 4 h for the conjugation of the antibodies and PS particles (Figure 3). Free-suspending antibodies were eventually washed away through centrifugation three times, and 1% bovine serum albumin (A2153, Sigma, St. Louis, MO, USA) was added to the particle suspension at 25 °C in a shaker for 1 h to block nonspecific binding.

#### 2.3.2. Functionalization of Probe Particles

Probe particles were obtained by covalently conjugating the probe antibody, rabbit polyclonal anti-LCN-1 IgG (17900-1-AP, Proteintech, Rosemont, IL, USA), to AuNPs according to the protocol of the gold conjugation kit (ab154873, Abcam, Cambridge, UK). To this end, the probe antibody solution was diluted to 0.1 mg/mL with the gold antibody diluent provided in the kit. Afterward, 12 µL of diluted antibody solution was thoroughly mixed with 42 µL of gold reaction buffer. The mixed solution was then added to the AuNP suspension to enable conjugation in a shaker at 400 rpm for 20 min. Finally, 5 µL of gold quencher was added to the mixture for the complete conjugation of the antibodies and AuNPs. The antibody-conjugated AuNPs were stored at 4 °C for subsequent use.

#### 2.3.3. Formation of Sandwiched Immunocomplex

The sandwiched immunocomplex was synthesized by mixing 20 µL of antibody-conjugated capture particles with 10 µL of the desired concentration of LCN-1 (a76611, Abcam, Cambridge, UK), incubating at 25 °C in a shaker for 15 min, and mixing with 15 µL of antibody-conjugated AuNPs. The formation of sandwiched immunocomplex eventually resulted in the colorimetric aggregation of probe particles because they were blocked along with large capture particles by the porous hydrogel.

### 2.4. Measurement Platform

For visual observation, the capillary-based detection device (i.e., a capillary filled with a porous hydrogel and a sponge plug) was sufficient to provide qualitative information. However, for quantitative analysis, additional components, including a backlight source, a detection box, and a smartphone, were required. The backlight was generated from the LCD screen of a laptop (Pavilion 15-au109TX, HP). A capillary tube holder and a reference capillary tube were placed inside the detection box, and an optical zoom lens (0.67×) having a working distance of 3.9 cm, was clamped on the camera of a smartphone to facilitate closeup imaging. In the LCN-1 detection process, a modified capillary was dipped into a vial containing the immunocomplexed particle solution until it reached the mark on the capillary. Subsequently, the capillary was flipped to stand vertically and allow the sandwiched immunocomplex solution to flow freely downward until the solution passed through the hydrogel. The capillary was then inserted into the capillary tube holder in the detection box, and the backlight was adjusted to 20% of full grayscale intensity (standard for reference) for the best and optimal image in detection (Supplementary Material Section S2 and Figures S3 and S4). Colorimetric aggregation inside the capillary was captured using a smartphone. The region of interest (ROI) was the particle aggregate in front of the porous hydrogel. The captured images were then quantitatively analyzed using ImageJ (https://imagej.net/Fiji). Finally, the concentration of the biomarkers was determined by analyzing the grayscale intensity of the aggregated particles in the image. Notably, the reference capillary tube in the study served as a standard when a different backlight source or smartphone was used. For instance, an intensity of the ROI in the reference capillary tube higher or lower than the standard would need to be readjusted to the same value before measurement could proceed.

### 2.5. Statistical Analysis

All data were obtained from independent tests and presented as mean ± standard deviation (SD). Comparison between the values was performed using student’s *t*-test. The significance level was set at 95% (*p* < 0.05) and the GraphPad Prism 5.0 software package (GraphPad Software Inc., San Diego, CA, USA) was used for data analysis.

## 3. Results and Discussion

### 3.1. Formation of Porous Hydrogel

**Figure 4:**
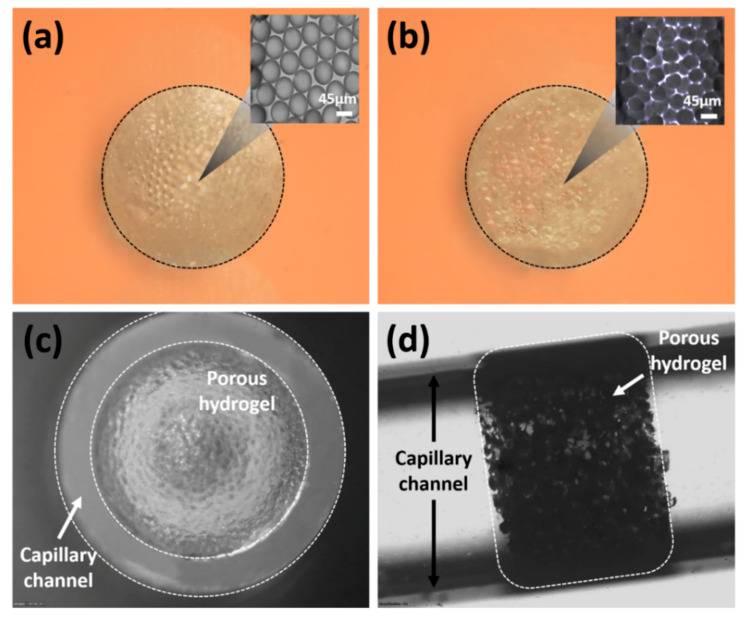
Microscope image of porous hydrogel (image scale 500 µm): (**a**) hydrogel filled the spaces of the self-assembled particles and closely-packed colloidal structure; (**b**) porous hydrogel after the removal of the template and the microstructure of the porous hydrogel; (**c**) frontal view of the porous hydrogel inside the capillary channel; (**d**) side view of the porous hydrogel inside the capillary channel.

A porous PEGDA hydrogel was obtained after completely dissolving the 45 μm PS particles with toluene. The fabrication process and the appearance of the porous hydrogel were observed with a microscope (Olympus, IX71, Tokyo, Japan). First, the self-assembled PS particles formed a hexagonal close-packed structure after evaporation (Figure 4a). Notably, evaporation is commonly used in promoting self-assembly for colloidal suspensions due to its simplicity and high reliability [33]. However, rapid evaporation may impair the order of self-assembly [34]. To avoid this unexpected disorder, the capillary tube was used for the self-assembly of particles. For 5 μL of colloidal suspension, the evaporation took 4 h to complete in a ventilated hood. A PEGDA solution was prepared and poured into the capillary tube to fill the interstitial space within the self-assembled structure. After the PS particles were removed with toluene, a raw porous PEGDA hydrogel was obtained (Figure 4b). The cross-sectional image of the porous hydrogel showed that the pore size remained close to the original template. The useful porous hydrogel section was obtained by cutting the capillary tube and transferring the remaining hydrogel to a new capillary tube. The front and side images of the transferred hydrogel inside the capillary tube were examined. The capillary tube was completely filled with the porous hydrogel (Figure 4c,d), indicating the successful fabrication of the diagnostic capillary.

### 3.2. Sandwiched Immunocomplex Detection

**Figure 5:**
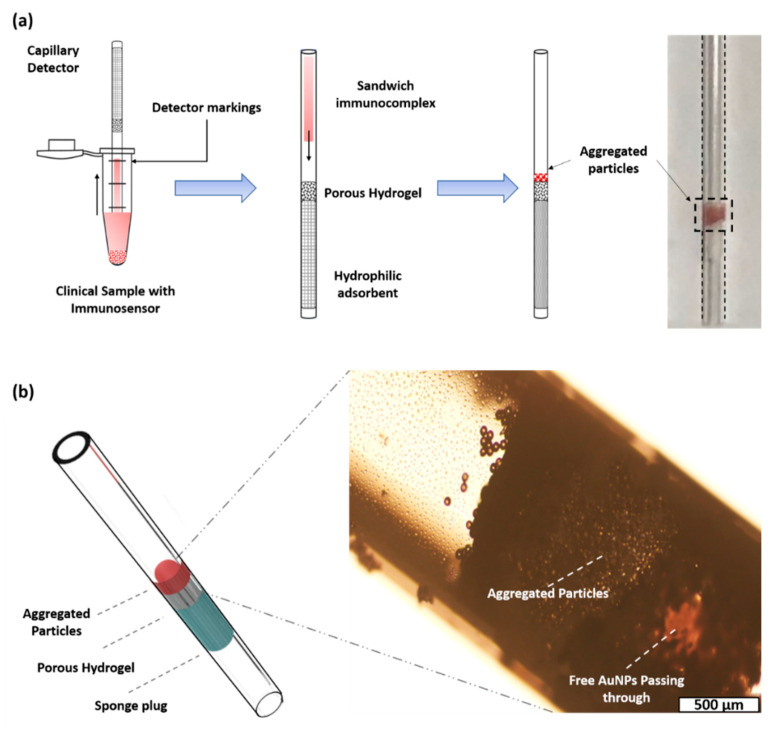
(**a**) Sandwiched immunocomplex detection process; (**b**) 3D model of capillary detector and aggregation of immunocomplex.

In our design, the capture and probe particles formed a sandwiched immunocomplex in the presence of LCN-1, resulting in colorimetric aggregation. By dipping the diagnostic capillary into the immunocomplexed solution and reaching a depth defined by a mark, a certain volume of immunocomplexed solution was dragged into the capillary tube through capillary force. When an appropriate amount of the solution was held in the tube, the capillary was then flipped over such that the sample would fall through the porous hydrogel through gravitational force (Figure 5a). When the sample reached the sponge plug at the other end of the porous hydrogel, powerful suction forced the remaining immunocomplexed solution to move forward until it dried out. The porous hydrogel served as a semipermeable barrier, and the sensing mechanism was realized by blocking the sandwiched immunocomplex to form colorimetric aggregation (Figure 5b). In the absence of target antigens, only plain capture particles stacked up, but free-suspending probe particles (AuNPs) passed through the porous hydrogel, leaving no noticeable emergence of color. Conversely, the presence of target antigens in the sample induced a sandwich formation on the immunocomplex. When this sandwiched immunocomplex was run through the capillary, they were blocked by the porous hydrogel because of size sieving. The aggregation of the sandwiched immunocomplex then produced a dark red color band because of stacked AuNPs, indicating a positive response (Supplementary Material Section S3 and Figure S5). Surface plasmon resonance on the stacked AuNPs induced the red color. However, the red color turned dark as more AuNPs stacked up to deplete light intensity in the presence of more LCN-1.

### 3.3. Colorimetric Assessment for Direct Visualization

**Figure 6:**
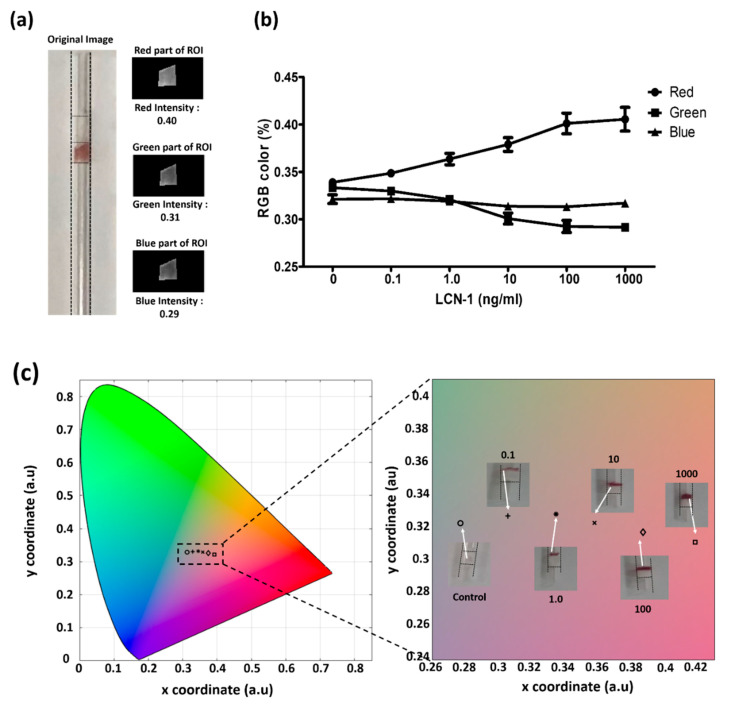
(**a**) MATLAB interface for image processing for color profile; (**b**) percentage of RGB of the sandwiched immunocomplex at different antigen concentrations; (**c**) CIE 1931 color profile of aggregated sandwiched immunocomplex for visualization with the naked eye. The unit of the numbers in the insets is ng/mL.

The images of the colorimetric aggregation were further recorded for the investigation of the relationship between color change and LCN-1 concentration. A ROI that covered the particle aggregate in the image was firstly selected. The ROI was then split into three output images according to RGB value (Figure 6a). These values were converted into (x, y) coordinates defining the color profile of the aggregated particles in the CIE 1931 chromaticity space. Through this process, the actual color of the aggregated sandwiched immunocomplex within the capillary device was determined. The RGB levels and color profiles of the sandwiched immunocomplex with six different concentrations of LCN-1 were plotted (Figure 6b). For the RGB variations in the immunocomplex at different antigen concentrations, the red value dramatically increased with antigen concentration. Notably, this color enhancement simply resulted from stacked AuNPs rather from the red shifting effect due to changes in the sizes of the aggregated AuNPs [35]. Furthermore, the CIE 1931 chromaticity space was used in quantitatively illustrating the color change resulting from the different concentrations of antigens (Figure 6c). Increase in antigen concentration led to increase in red intensity, which was the basis for macroscopic concentration reading.

### 3.4. Quantitative Assessment with the Measurement Platform

**Figure 7:**
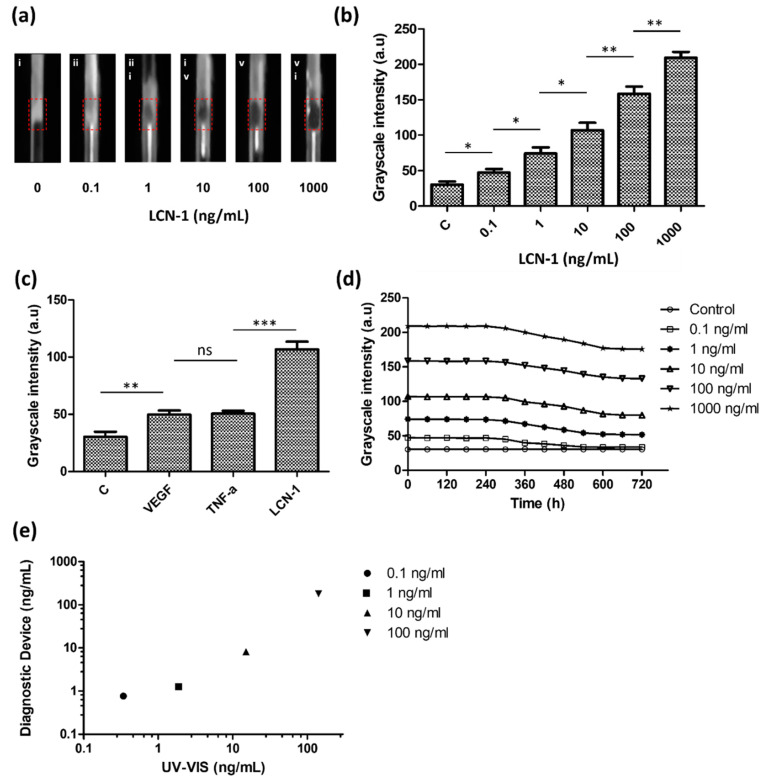
Quantitative assessment of the detection platform: (**a**) grayscale images of diagnostic tool inside the detection box; (**b**) calibration of the grayscale intensity of sandwiched immunocomplex with respect to different lipocalin-1 (LCN-1) concentrations ranging from 100 pg/mL to 1 µg/mL; (**c**) binding specificity of the capture and probe particles in the presence of different antigens; (**d**) binding stability of sandwiched immunocomplex with respect to different times; (**e**) reliability of the detection platform. Comparison of LCN-1 concentration detection results between the proposed diagnostic tool and spectrophotometer. The symbols “*”, “**”, “***”, “ns” denote *p* < 0.05, *p* < 0.01, *p* < 0.001, and *p* > 0.05, respectively, under student’s *t* test (*n* = 5). The error bars represent standard deviation.

In addition to direct visualization, the capillary device was also evaluated through quantitative analysis. Unlike the previous color mode that showed potential for quick screening by direct visualization, a grayscale mode exhibited a linear relationship between the light intensity and protein concentration. Given the background noise resulting from different measurement conditions, the capillary device was measured in a customized detection box. The detection box provided a dark environment with a controllable light source supplied from the laptop screen. For quantitative imaging, a smartphone was connected to an optical lens holder and attached to the detection box. Images recorded by the smartphone were converted into grayscale, and their average ROI intensity was calculated (Figure 7a). Grayscale intensity was obtained over a concentration ranging from 100 pg/mL to 1 μg/mL, covering three orders of magnitude. The results indicated that a high antigen concentration increased the quantity of the sandwiched immunocomplex aggregates and grayscale intensity. Based on the intersection of the threefold error bars of the control and linear trend line, the estimated LOD level for the target biomarker was 1 ng/mL. (Figure 7b).

For the investigation of binding specificity, two irrelevant antigens, VEGF and TNF-α, were used in the study. All of the three antigens, including LCN-1, were prepared (10 ng/mL) and separately mixed with the capture and probe particles. According to their grayscale intensities (Figure 7c), although VEGF and TNF-α seemed to induce some nonspecific bindings, they both showed lower immune responses than LCN-1. The higher grayscale intensity of LCN-1 as compared with the intensities of the other two antigens showed that the current capture and probe particles were specifically responsive to LCN-1.

Another experiment was performed to investigate the stability of the sandwiched immunocomplex. The grayscale intensities of the five different concentrations of the LCN-1 control samples were continuously monitored for over 720 min (Figure 7d). Notably, the grayscale intensities of all the samples appeared to have maintained their levels in the first 300 min and then gradually decreased over time afterward. This trend was likely due to the degradation of the antibody in a moderate environment, specifically at room temperature, which led to disintegration of capture and probe particles. Accordingly, it was recommended to complete the color reading within 5 h after the samples were injected into the capillary tube.

Lastly, LCN-1 was also measured using a UV−VIS spectrophotometer (NanoDrop One, Thermo Scientific) for validation. The protein concentration was determined by the absorbance peak at 280 nm according to the instrument standard protocol. A calibration curve (i.e., absorbance vs. protein concentration) from 30 pg/mL to 3 mg/mL using bovine serum albumin was established. Subsequently, four control samples with LCN-1 concentration ranging from 100 pg/mL to 100 ng/mL were prepared and then separately measured with the spectrophotometer and our diagnostic capillary (Figure 7e). When spectrophotometric results were used herein as reference values, the accuracy of the proposed diagnostic device (Equation (1)) varied between 44.28% (0.1 ng/mL of antigen) and 79% (100 ng/mL of antigen), implying that the current diagnostic device remained incapable of low concentrations below 1 ng/mL. For diseases with rich biomarkers, such as LCN-1 in DR, however, this device is suitable as a rapid screening tool and can provide valuable information about the presence of biomarkers in samples.

(1) $$\mathrm{Accuracy}\;\left(\%\right)=\left(1-\left|\frac{\mathrm{diagnostic}\;\mathrm{device}\;-\;\mathrm{macroscale}\;\mathrm{method}}{\mathrm{macroscale}\;\mathrm{method}}\right|\right)\times 100\;$$

### 3.5. Detection of Biomarker LCN 1 in Clinical Tear Samples

**Figure 8:**
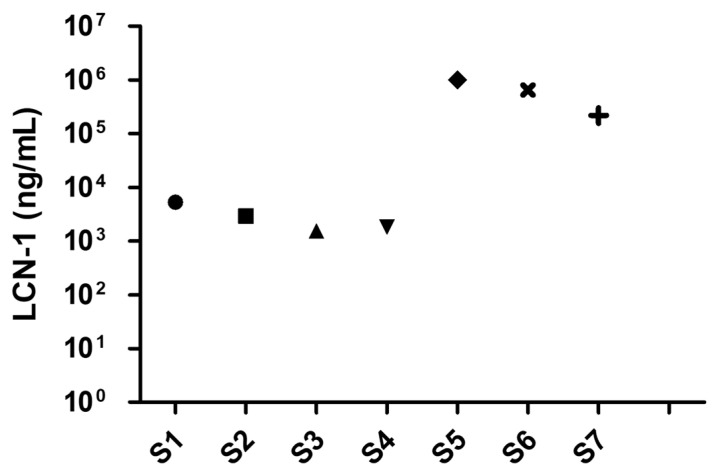
Preclinical test with four healthy subjects (**S1**–**S4**), two (**S5**, **S6**) clinically diagnosed proliferative diabetic retinopathy (PDR) patients, and one (**S7**) diagnosed nonproliferative diabetic retinopathy (NPDR) patient.

The practicability of the diagnostic capillary device was evaluated according to the measured concentration of the DR biomarker LCN-1 in tear samples collected from seven clinical volunteers. The volunteers included four healthy subjects and three DR patients. Tear sample collection was conducted by a medical specialist at the Department of Ophthalmology, National Cheng Kung University Hospital (NCKUH) in compliance with the IRB agreement # A-ER-105-113. The patients were initially preselected by the ophthalmologist. The exclusion criteria included dry eye syndrome, macular degeneration, and under drug treatments. Approximately 10 µL of tear fluid was extracted from the lower eyelid of each volunteer through a sterilized glass tube. To this end, the lower eyelid was firstly pulled downward, revealing the conjunctival sac into which the glass tube was held horizontally. Owing to the capillary effect, the tear fluid then automatically flowed into the glass tube. When sufficient tear fluid was collected, the tear sample was pipetted out of the glass tube and injected into a microcentrifuge tube. The tube was stored in a refrigerator at −70 °C for subsequent use. Notably, unlike in the previous calibration steps, the clinical samples were diluted with 1× PBS + 0.1% Tween 20 (PBST) 100-fold. This step ensured that LCN-1 level was within the dynamic range of measurement (1 pg/mL–1 µg/mL). Afterward, the final LCN-1 concentration was obtained by multiplying the measured data 100-fold. Among these volunteers, four healthy subjects (S1–S4) were diagnosed with no clinical history of diabetes or any other reported eye-related diseases, two (S5, S6) were clinically confirmed as PDR patients, and one (S7) was a NPDR patient. The LCN-1 concentrations of the seven clinical samples were obtained using the proposed capillary device (Figure 8). As expected, the healthy subjects appeared to show lower concentrations than the other PDR and NPDR patients. However, the measured LCN-1 concentrations in all the samples were lower than the previously reported threshold of LCN-1 (1–2 mg/mL) [36] in healthy tear fluids. The dramatic deviations were likely due to the multiple dilutions performed during sample preparation. The original concentration was not linearly proportional to the dilution ratio. Nevertheless, the significant LCN-1 level differences between the healthy subjects and the patients with PDR or NPDR implied that potential patients with PDR or NPDR may be diagnosed accurately with the proposed diagnostic tool. This diagnostic device provides a rapid screening tool for performing preventive DR treatments in the early stage.

## 4. Conclusions

This study presents a simple method for detecting small molecules or low-abundance biomarkers with a modified capillary tube and a portable detection platform for color profiling. In the proposed detection method, the capillary tube was modified by embedding a porous hydrogel, which served as a filter and sponge plug to drag liquid samples to accelerate detection. In addition, capture and probes particles were conjugated with selected antibodies. In the presence of antigen, the two particles would be linked together forming a sandwiched immunocomplex. In the detection process, the capillary tube was submerged into the vial containing the sandwiched immunocomplex up to the markings. Through capillary force, the immunocomplex was dragged into the capillary tube, and upon sucking enough sample, the detector was flipped over for additional gravitational force. When the sample reached the sponge plug on the other end of the porous hydrogel, powerful suction kept dragging the remaining immunocomplex solution to move forward until it dried out. The porous hydrogel served as a semipermeable barrier and its sensing mechanism was dependent on blocking sandwiched immunocomplex particles to form color. In the absence of target antigens, only plain capture particles stacked up, and free-suspending probe particles passed through the porous hydrogel, and thus no noticeable color was observed. The aggregated immunocomplex was observed and quantified by the grayscale intensity of an image. The LOD of the technique reached as low as 1 ng/mL. The evaluation results revealed that accuracy was as high as 79% and high binding specificity to the target biomarker LCN-1. The suitability of the proposed detection system for real-world detection applications was investigated by measuring LCN-1 in tear samples collected from volunteers. Significant differences in the LCN-1 level between the healthy subjects and patients with PDR or NPDR implied that potential patients with PDR or NPDR may be accurately diagnosed with the proposed diagnostic tool. Finally, this diagnostic device provides a low-cost (~1 USD) and rapid screening tool (1 min of colorimetry) for preventive DR treatments in the early stages.

## Supplementary Materials

The following are available online at https://www.mdpi.com/2079-6374/10/10/130/s1. Figure S1. Porous hydrogel fabrication: PS particles removal. Figure S2. Porous hydrogel relocation to new capillary tube. Figure S3. Backlight Optimization. Figure S4. (a) Image taken inside the detection box. (b) Actual experimental set-up of detection platform. Figure S5. Porous hydrogel relocation to new capillary tube.
