# Supplementary material for: Colorimetric Diagnostic Capillary Enabled by Size Sieving in a Porous Hydrogel
Source: Biosensors (Basel). 2020 Sep 23;10(10):130. doi: 10.3390/bios10100130 (PMC7598291; doi:10.3390/bios10100130)
Supplement: Supplementary file 1 [file biosensors-10-00130-s001.pdf]

Colorimetric Diagnostic Capillary Enabled by Size Sieving in a Porous Hydrogel

John Mello Camille C. Guzman[1], Sheng-Min Hsu[2], and Han-Sheng Chuang[1, 3, *]

[1] Department of Biomedical Engineering, National Cheng Kung University, Tainan 701, Taiwan

[2] Department of Ophthalmology, National Cheng Kung University Hospital, Tainan 701, Taiwan

[3] Center for Micro/Nano Science and Technology, National Cheng Kung University, Tainan 701, Taiwan

Corresponding author: oswaldchuang@mail.ncku.edu.tw

Supplementary Material Section 1

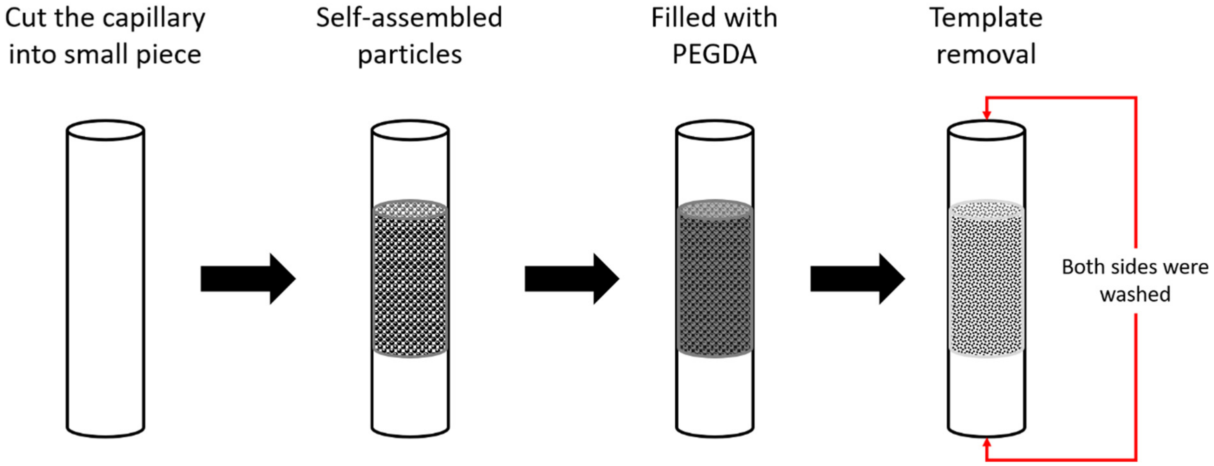

Figure S1. Porous hydrogel fabrication: PS particles removal.

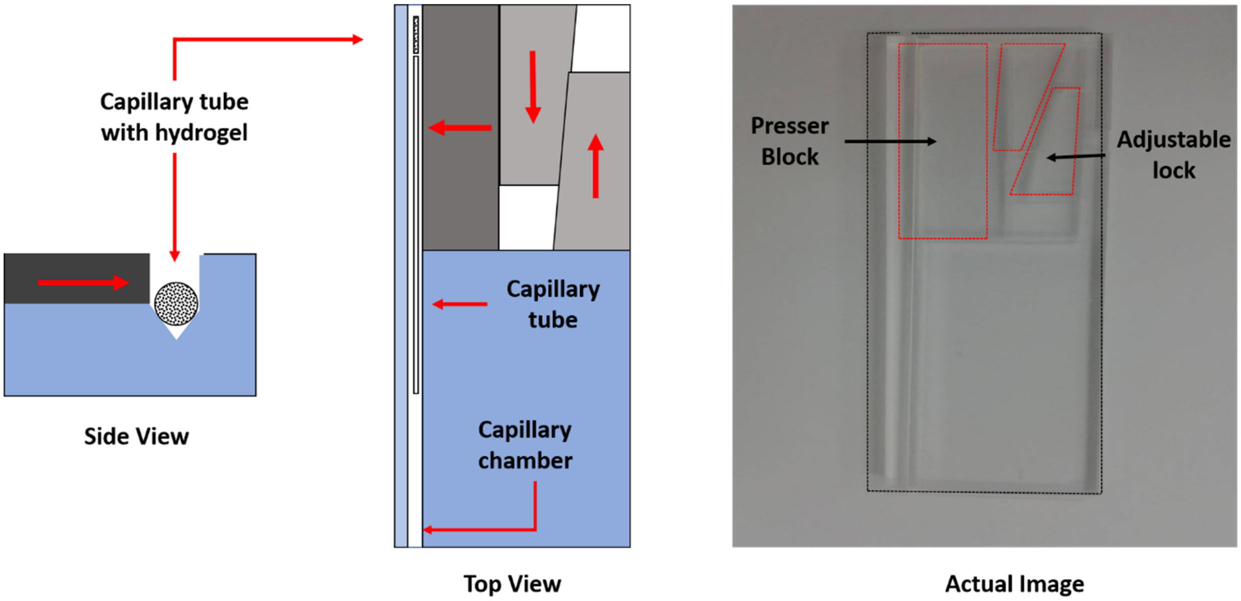

Figure S2. Porous hydrogel relocation to new capillary tube.

Supplementary Material Section 2

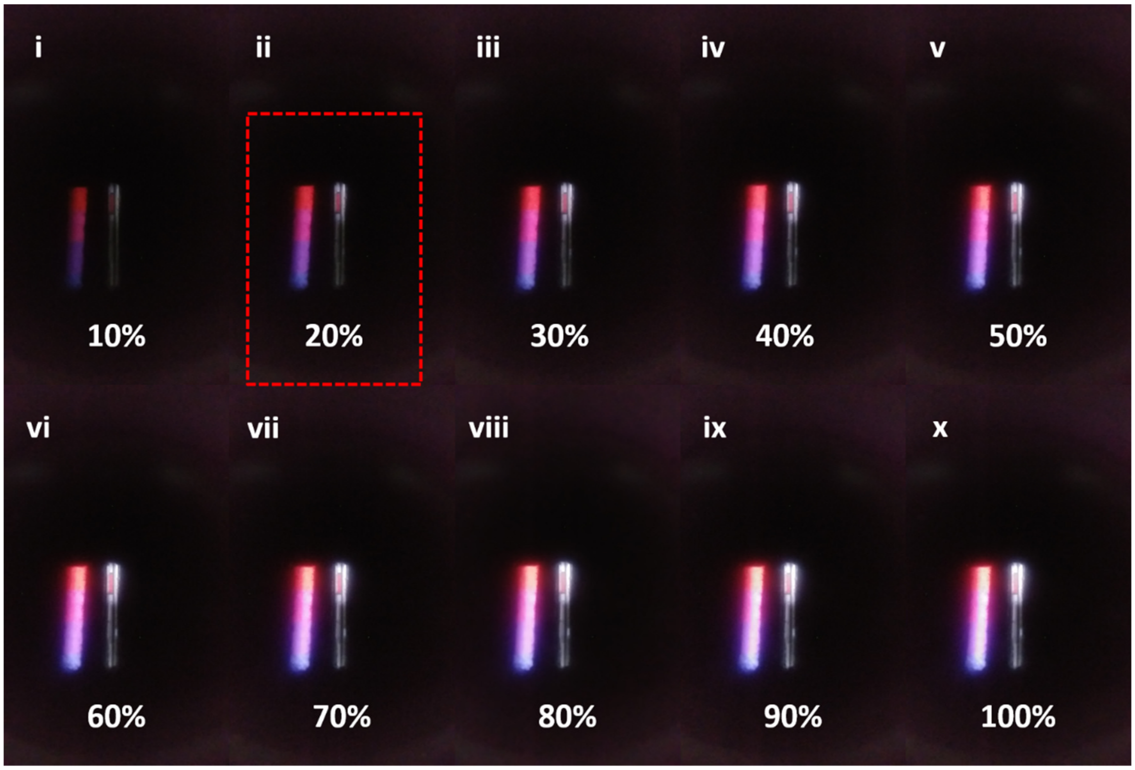

Figure S3. Backlight Optimization.

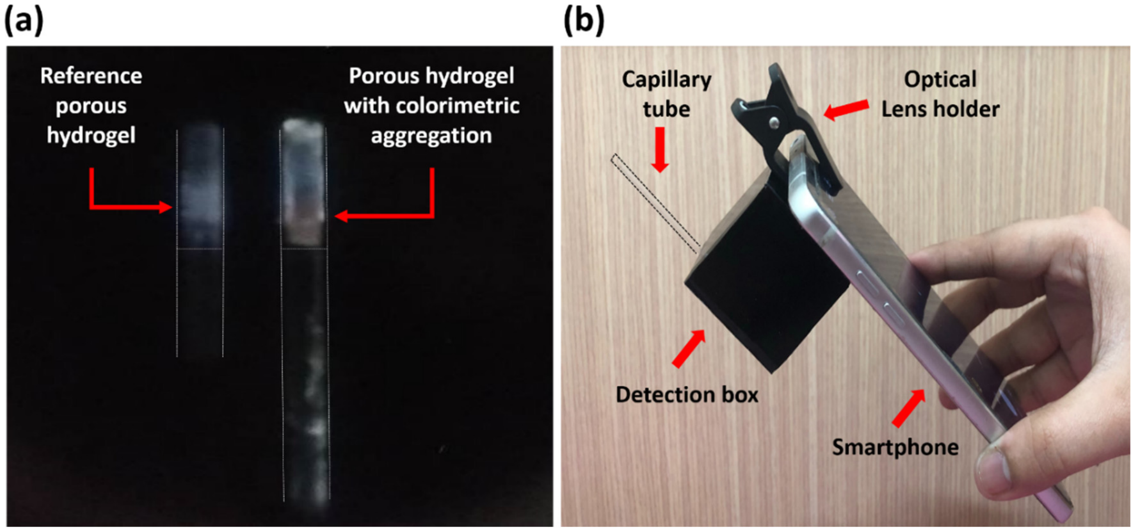

Figure S4. (a) Image taken inside the detection box. (b) Actual experimental set-up of detection platform.

Supplementary Material Section 3

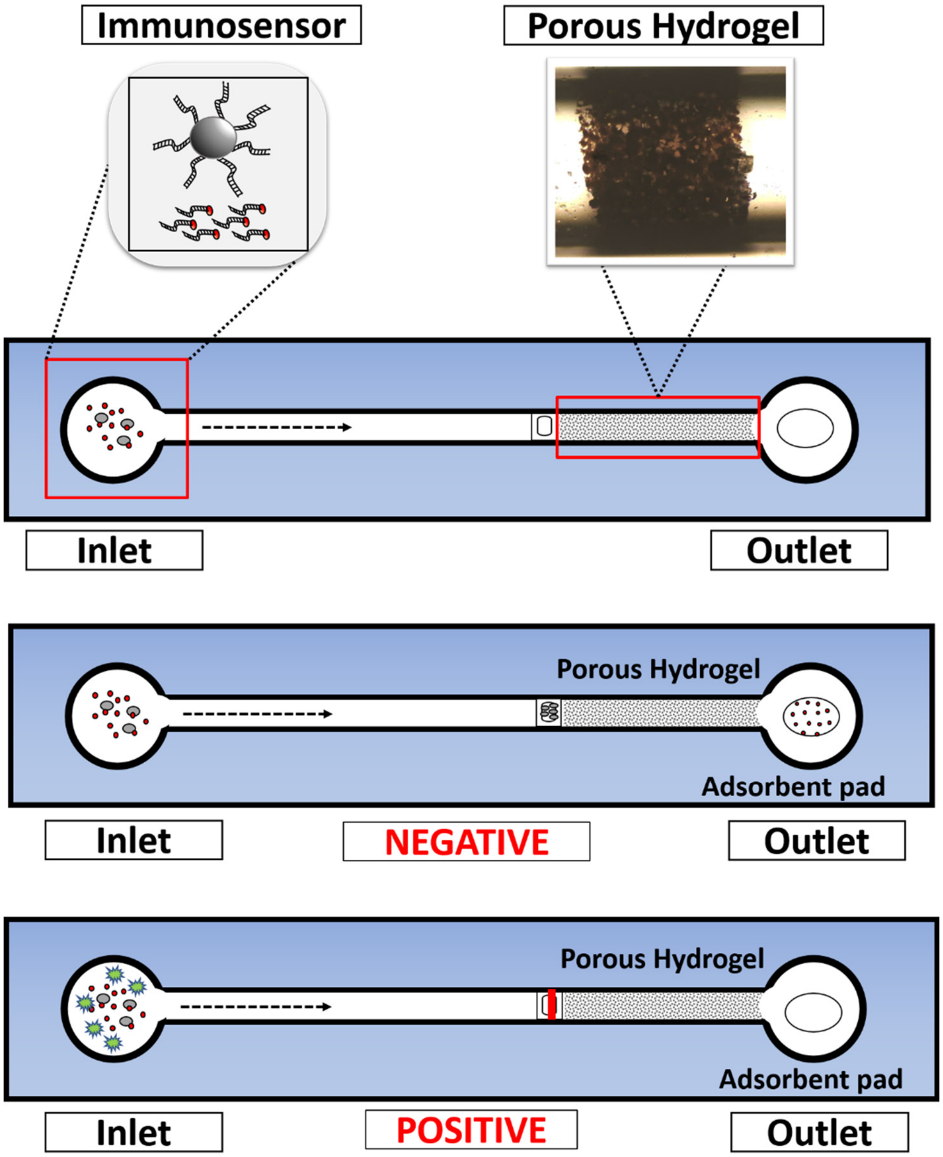

Figure S5. Porous hydrogel relocation to new capillary tube.
